# Suprascapular Neuropathy in Collegiate Tennis Player: A Case Report

**DOI:** 10.7759/cureus.20824

**Published:** 2021-12-30

**Authors:** Clayton R Walker, J Christian Y Belisario, John M Vasudevan

**Affiliations:** 1 Physical Medicine and Rehabilitation, University of Pennsylvania, Philadelphia, USA

**Keywords:** rotator cuff pathology, infraspinatus atrophy, physiatry, electromyography, orthopaedics and sports physical therapy, sports medicine, shoulder pathology, collegiate athlete, tennis, suprascapular neuropathy

## Abstract

Suprascapular neuropathy can be seen in overhead athletes and should be considered when evaluating an overhead athlete presenting with shoulder pain and/or weakness. The suprascapular nerve innervates the supraspinatus and the infraspinatus, but dynamic entrapment as it passes under the spinoglenoid ligament at the spinoglenoid notch can lead to isolated denervation of the infraspinatus. Specific movements common in tennis cause tensioning and compression of the spinoglenoid ligament and may predispose players to dynamic entrapment at this location. We present a case of an 18-year-old male collegiate tennis player presenting with suprascapular neuropathy causing isolated denervation of the infraspinatus. This case illustrates the consequences of noncompliance with physical therapy and serves as a review of the pathophysiology, evaluation, and treatment of suprascapular neuropathy.

## Introduction

Suprascapular neuropathy (SSN) is a rare condition and is more commonly seen in overhead athletes due to repetitive movements at the shoulder [1-6]. The suprascapular nerve is a mixed motor and sensory nerve that originates from the upper trunk of the brachial plexus with contributions from C5, C6, and occasionally C4 nerve roots [1,6]. It travels through the posterior cervical triangle to the superior border of the scapula, where it passes through the suprascapular notch and under the superior transverse scapular ligament [6]. The nerve then travels laterally along the supraspinous fossa, where it branches to provide motor innervation to the supraspinatus and receive sensory input from the acromioclavicular and glenohumeral joints [6]. The nerve continues along the supraspinous fossa until it reaches the scapular spine, where it passes through the spinoglenoid notch and under the spinoglenoid ligament [6]. It then proceeds inferiorly to provide motor innervation to the infraspinatus [6]. Patients with SSN will present with weakness with abduction and external rotation of the shoulder, and dull pain localized to the superior posterolateral aspect of the shoulder [1]. We present a National Collegiate Athletic Association (NCAA) Division I male tennis player with SSN who originally presented with chronic shoulder discomfort and isolated infraspinatus weakness and atrophy. This case illustrates the consequences of noncompliance with physical therapy (PT) and serves as a review of the pathophysiology, evaluation, and treatment of SSN.

## Case presentation

A right-handed 18-year-old male tennis player presented to a primary care sports medicine clinic for evaluation of right shoulder pain and weakness at the start of the season. He had noticed a progressive decline in performance within the past year, which ultimately prompted him to be evaluated. His symptoms were localized to the right posterolateral shoulder girdle, which he described as mild, dull muscle soreness. His symptoms were exacerbated with tennis play and improved with rest. He endorsed mild weakness with external rotation without sensory loss. Initial examination revealed decreased strength with external rotation of the right shoulder (Manual Motor Testing: 3/5), moderate atrophy of his right infraspinatus, and preservation of the supraspinatus. There was no point tenderness, no sensory deficit, and he had full active and passive range of motion of the shoulder without instability.

A shoulder X-ray was done and was unremarkable. Electromyography (EMG) and nerve conduction studies (NCS) demonstrated normal NCS, but EMG was significant for abnormal spontaneous activity and neuropathic changes limited to the infraspinatus with preservation of the supraspinatus, which indicated a chronic SSN localized to the spinoglenoid notch. Magnetic resonance (MR) arthrogram was negative for compressive pathology and showed an intact labrum and rotator cuff. Since there was no anatomic anomaly attributed to the patient's symptoms, he was referred for PT.

**Figure 1:**
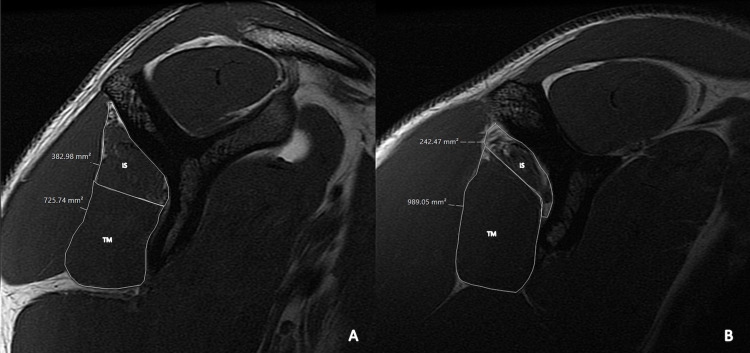
Sagittal T1-weighted MRI of the right shoulder with the area calculated for teres minor (TM) and infraspinatus (IS) from 2019 (A) and 2021 (B).

Due to the COVID-19 pandemic, he was unable to stay on campus and was lost to follow-up. He re-presented 15 months later and had not completed any formal PT but had continued to practice and play tennis. His exam was notable for markedly increased atrophy of the infraspinatus and stable weakness with external rotation (Manual Motor Testing: 3/5), so he was referred to orthopedic surgery. A repeat MR arthrogram showed severe atrophy of the right infraspinatus and marked hypertrophy of the teres minor when compared to the imaging obtained 15 months prior (Figure 1). Repeat EMG demonstrated progression of abnormal spontaneous activity and neuropathic changes in the infraspinatus with continued preservation of the supraspinatus. Due to the extent of the atrophy, the orthopedic surgeon did not think it would recover with a decompressive surgery. The patient returned to full team activities without restriction but with careful monitoring.

## Discussion

Suprascapular nerve entrapment is an uncommon cause of shoulder pain but is more common in overhead athletes. The true prevalence is unknown, but estimates made from case series in athletes estimate the prevalence to be as high as 12-33% in athletes compared to 1-2% reported in the general population [1-5]. Left untreated, suprascapular nerve entrapment may lead to neuropathy and irreversible infraspinatus and/or supraspinatus weakness; accurate diagnosis and treatment may prevent such consequences [7].

Suprascapular nerve entrapment may occur at either the suprascapular or spinoglenoid notch [8]. Secondary entrapment can occur due to space-occupying lesions such as ganglion cysts, neoplasms, or ossified scapular ligaments. Traumatic or post-traumatic etiologies including large, retracted rotator cuff tears, scapular fractures, penetrating traumas, or hematomas, can also compress the suprascapular nerve [1].

As the suprascapular nerve travels through the spinoglenoid notch, it passes underneath the spinoglenoid ligament making it susceptible to dynamic entrapment [9,10]. Shoulder abduction and external rotation cause compression at the spinoglenoid notch, while internal rotation and cross-body adduction cause tightening of the spinoglenoid ligament [10,11]. Tennis maneuvers require these motions multiple times per match. For example, in the late motion of the serve and in the forehand, torque is generated by shoulder internal rotation, cross-body adduction, and elbow extension [12,13]. Torque in the backhand swing is conversely generated by shoulder external rotation, abduction, and extension [14]. These repetitive movements can predispose an athlete to dynamic entrapment of the suprascapular nerve and subsequent suprascapular neuropathy.

Typically, a patient with SSN presents with dull, chronic pain in the superior posterolateral aspect of the shoulder with possible radiation into the neck or lateral aspect of the arm, and weakness with shoulder abduction and external rotation [15]. Workup includes shoulder radiographs and MR imaging, but the gold standard for diagnosis is EMG and NCS [11,15]. If entrapment occurs at the suprascapular notch, denervation will be evident in both the supraspinatus and the infraspinatus. If entrapment occurs at the spinoglenoid notch, denervation will only be observed in the infraspinatus. Treatment initially involves activity modification, pain control, and a PT program. Indications for surgery include failure of nonoperative treatment or the presence of a reversible anatomic space-occupying lesion [1,11].

PT should focus on activity modification, strengthening of the deltoid, the rotator cuff muscles, and strengthening scapular stabilizers including the serratus anterior, levator scapulae, trapezius, and rhomboids, as well as neuromuscular facilitation exercises. A study that incorporated the above therapy in 12 patients with isolated suprascapular nerve pathology had good to excellent results in all 12 patients with normal EMG and NCS after completing the six-month treatment protocol [16].

The patient’s collegiate team uses an injury surveillance and prevention system (Sparta Science), which assesses kinematic variables from vertical jump, single-arm plank, and blindfolded single-leg balance testing; this information is used to flag athletes who are at higher risk of sustaining an injury, which guides intervention. Previous studies have evaluated the efficacy of such systems and saw a 30% reduction in the number of surgeries athletes required with this system [17]. Our patient was able to hold a left-arm plank for 57 seconds (team average 57 seconds) but could only hold a right arm plank for 43 seconds (team average 53 seconds), which may have been related to decreased rotator cuff stability secondary to infraspinatus weakness. Although this was not the reason he presented to the clinic, it does provide some evidence that these types of systems can be used to screen for pathologic muscle imbalance.

Our patient failed to complete a formal PT program and as a result, he had persistent evidence of denervation on EMG and progressive, irreversible atrophy seen on MRI. Although it is impossible to say for certain that PT would have prevented this outcome, PT has been previously shown to be an effective treatment for SSN [16]. It is notable, however, that his strength remained stable throughout the 15 months of follow-up even though his infraspinatus atrophy worsened. This may be due to compensation by other external rotators of the shoulder, which is supported by the marked hypertrophy of the teres minor seen on follow-up imaging (Figure 1). Previous research has shown that the teres minor and supraspinatus contribute 30% of external rotation strength at baseline, so it’s not unreasonable to think that hypertrophy of these two muscles over time can lead to better compensation [18].

## Conclusions

This case report highlights the importance of compliance with physical therapy when treating SSN by showing the potential consequences of continuing sports participation without addressing the underlying pathology. The patient developed irreversible atrophy of his infraspinatus but preserved some strength through hypertrophy of other external rotators allowing him to continue to play tennis at the NCAA Division I level. Although he complained of a decline in performance on presentation, continued follow-up will be needed to assess if these compensatory mechanisms can completely compensate for his infraspinatus weakness.
